# Pre-Harvest Agronomic Reduction in *Fusarium* Mycotoxins in Winter Barley: Effects of Agrotechnical Intensity on Grain Mycobiome, DON/ZEN and Feed-Quality Traits

**DOI:** 10.3390/toxins18040171

**Published:** 2026-04-02

**Authors:** Sylwia Barbara Okorska, Magdalena Serafin-Andrzejewska, Agnieszka Pszczółkowska, Agnieszka Falkiewicz, Marcin Włodarczyk, Mengcen Wang, Adam Okorski, Marcin Kozak

**Affiliations:** 1 Department of Genetics and Plant Pathophysiology, Faculty of Agriculture and Forestry, University of Warmia and Mazury in Olsztyn, Prawocheńskiego 21, 10-727 Olsztyn, Poland; sylwia.okorska@uwm.edu.pl (S.B.O.); agnieszka.pszczolkowska@uwm.edu.pl (A.P.); 2 Institute of Agroecology and Plant Production, Wrocław University of Environmental and Life Sciences, Grunwaldzki Sq. 24 A, 50-363 Wrocław, Poland; agnieszka.falkiewicz@upwr.edu.pl (A.F.); marcin.kozak@upwr.edu.pl (M.K.); 3 Research Centre for Cultivar Testing, Experimental Station for Cultivar Testing in Zybiszów, Zybiszów 1, 55-080 Zybiszów, Poland; m.wlodarczyk@zybiszow.coboru.gov.pl; 4 State Key Laboratory of Rice Biology and Breeding, Ministry of Agricultural and Rural Affairs, Laboratory of Molecular Biology of Crop Pathogens and Insects Pests, Zhejiang University, Hangzhou 310058, China; wmctz@zju.edu.cn; 5 Zhejiang Key Laboratory of Biology and Ecological Regulation of Crop Pathogens and Insects, Zhejiang Engineering Research Center for Biological Control of Crop Pathogens and Insect Pests, Institute of Pesticide and Environmental Toxicology, Zhejiang University, Hangzhou 310058, China

**Keywords:** *Hordeum vulgare*, food and feed safety, quality, yield, *Fusarium*, DON, ZEN

## Abstract

Winter barley (*Hordeum vulgare* L.) is an important crop used for feed, food, malting, and bioethanol production. Recent research indicates that the seed mycobiome significantly influences seed health and usability, affecting its potential applications. This study examined the fungal species present in seven barley cultivars grown under two agrotechnical regimes. Fungal species were classified according to their effects on seeds and plants, and biodiversity indices were calculated for each group. Enhanced agrotechnical practices increased yields and improved grain quality. Higher DON concentrations were observed in low-yield treatments. Mycological analysis revealed that field fungi, particularly *Fusarium*, dominated the grain mycobiome and were associated with infection and reduced seed quality. High Dominance (Y), Margalef, and Shannon–Wiener indices for quality-deteriorating fungi correlated with lower yields, while the Dominance index (λ) for these fungi was negatively correlated with grain protein content. The prevalence of specific fungi on seeds depends on storage conditions and fungal adaptations, which may result in complementary consortia. Understanding these interactions can support the development of effective seed storage strategies and inform material classification and future use decisions.

## 1. Introduction

Winter barley (*Hordeum vulgare* L.) is primarily cultivated for feed purposes, but it is also used as a raw material for the brewing and food industries [1]. In addition to its nutritional protein content, barley grain contains large amounts of dietary fibre—particularly soluble fractions such as β-glucans and arabinoxylans—as well as many important vitamins, including vitamin E and B-group vitamins [2]. Compared with spring barley, its yield is more stable and less dependent on climatic conditions, which is of great significance in the context of ongoing climate change [3]. The cultivars currently grown in Poland require 70–90 mm of rainfall during the autumn growing season. After the start of vegetation in spring, until harvest, the optimal amount of rainfall is 195 mm on medium soils and about 250 mm on lower-quality soils. Throughout the growing season, the sum of daily temperatures above 3 °C should be 1134 °C [4].

The longer growing period, more efficient water use, and higher number of grains contribute to greater productivity and yield stability of winter barley, resulting from key phenological phases occurring during periods of lower temperatures and higher water availability [5,6]. One of the main advantages of winter barley is its high drought tolerance, which largely results from a low transpiration coefficient (350) and early maturation [4,7].

The occurrence of barley diseases—particularly *Fusarium* head blight (FHB)—affects yield quantity, feed properties, and the suitability of the grain as an industrial raw material [8]. FHB is caused by fungi of the *Fusarium* genus, with *F*. *graminearum* being the predominant species under Polish climatic conditions [9]. Other species of lesser significance as FHB pathogens in Poland include *F. avenaceum*, *F. crookwellense*, *F. culmorum*, *F. equiseti*, *F. langsethiae*, *F. sporotrichioides*, *F. oxysporum*, *F. tricinctum*, and the commonly occurring but weakly pathogenic *F*. *poae* [10,11,12]. *Fusarium* fungi infect cereal spikes during flowering and subsequently develop within the grain, leading to contamination with mycotoxins [13].

From the flowering stage to grain filling, dynamic changes occur in the fungal profile that shape the wheat mycobiome [14]. A modern approach to plant protection integrates the influence of various factors, such as plant genotype [15] and agrotechnical conditions combined with control methods [16], in shaping a healthy microbiome colonising the spicosphere [17], which determines the reduction in mycotoxins from field crops to seed storage [18].

The occurrence of specific mycotoxins depends on the toxigenic capacity of *Fusarium* species, which varies not only among species but also among individual strains [19]. Deoxynivalenol (DON) and zearalenone (ZEN) are the two most frequently identified mycotoxins in cereal grain in Poland [20], produced mainly by *F. culmorum* and *F. graminearum* [21]. These toxic secondary metabolites pose a potential risk to food and feed safety, and their consumption can lead to various adverse health effects in both animals and humans.

DON, classified as a type B trichothecene, is also known as vomitoxin due to its ability to induce vomiting in pigs. Its presence in animal feed causes symptoms such as anorexia, vomiting, and diarrhoea, while severe cases may lead to circulatory system damage or even death [22,23]. Pigs are the most sensitive species to DON, exhibiting reduced feed intake, followed by gastritis, enteritis, necrosis, and haemorrhaging within the gastrointestinal tract [24].

ZEN exhibits oestrogenic properties and primarily affects the reproductive system. Its ingestion during pregnancy may cause abortions, stillbirths, and developmental abnormalities in pigs, and it can also adversely affect the central nervous system, leading to symptoms such as nausea, chills, headache, disorientation, and impaired coordination [25]. Gilts are particularly susceptible to ZEN, and typical symptoms of intoxication include inflammation of the vulva and vagina accompanied by abnormal mammary gland development, whereas boars exhibit decreased libido, testicular atrophy, and reduced semen quality [26].

Modern winter barley breeding programmes worldwide are largely focused on developing genotypes with enhanced yield potential, broad adaptability to changing climatic conditions and extreme weather events, and favourable responses to agronomic management practices [27].

Agronomic practices such as the cultivation of resistant genotypes, early sowing, crop rotation, and fungicide application can significantly reduce barley grain contamination with mycotoxins. Therefore, the implementation of integrated agronomic practices for the management of Fusarium head blight (FHB) and mycotoxin contamination is recommended to minimise the level of fertilisation in barley production [28,29]. Nitrogen fertilisation is one of the most common and indispensable agronomic measures used to achieve high yields.

However, there is limited information regarding the influence of fertilisation on mycotoxin occurrence in barley. For this reason, the aim of the present study was to determine the effect of cultivar and agrotechnical level, which integrated the use of two doses of nitrogen fertilisation, fungicides, growth regulators, and a multi-micronutrient foliar fertiliser, on the occurrence of mycotoxins in harvested winter barley grain.

An important new research direction is to determine how agrotechnical factors shape the communities of fungi colonising seeds immediately after harvest, which may in turn provide information on the role of the seed microbiome, whose species composition results from complex biological processes occurring in field conditions that influence crop formation.

Therefore, the objective of this study was to investigate the relationships between the integration of agronomic management, fungal biodiversity indices characterising the qualitative composition of the grain mycobiome, and grain quality parameters.

The agrotechnical level (AL) represents a complex management package because the basic level (A1) and intensive level (A2) differ simultaneously in nitrogen application, fungicide use, growth regulator use, and micronutrient fertilisation. This comprehensive approach was used in subsequent analyses to compare the impact of agrotechnology as a package of measures using variance analysis, Spearman’s rank correlation, and diversity indices across fungal categories.

## 2. Results

Statistical analysis showed that the individual components of yield structure and the concentrations of mycotoxins (DON and ZEN) in the grain of different winter barley cultivars were influenced by the experimental factors, namely cultivar and agrotechnical level, and were also dependent on the year of the study (Table 1). A detailed analysis of the main effects revealed no statistically significant influence of the agrotechnical level on vegetable fibre content or ZEN concentration. Furthermore, no significant differences were found in ZEN content between the 2019 and 2020 growing seasons (Table 1).

### 2.1. Yield Structure Components

**Figure 1:**
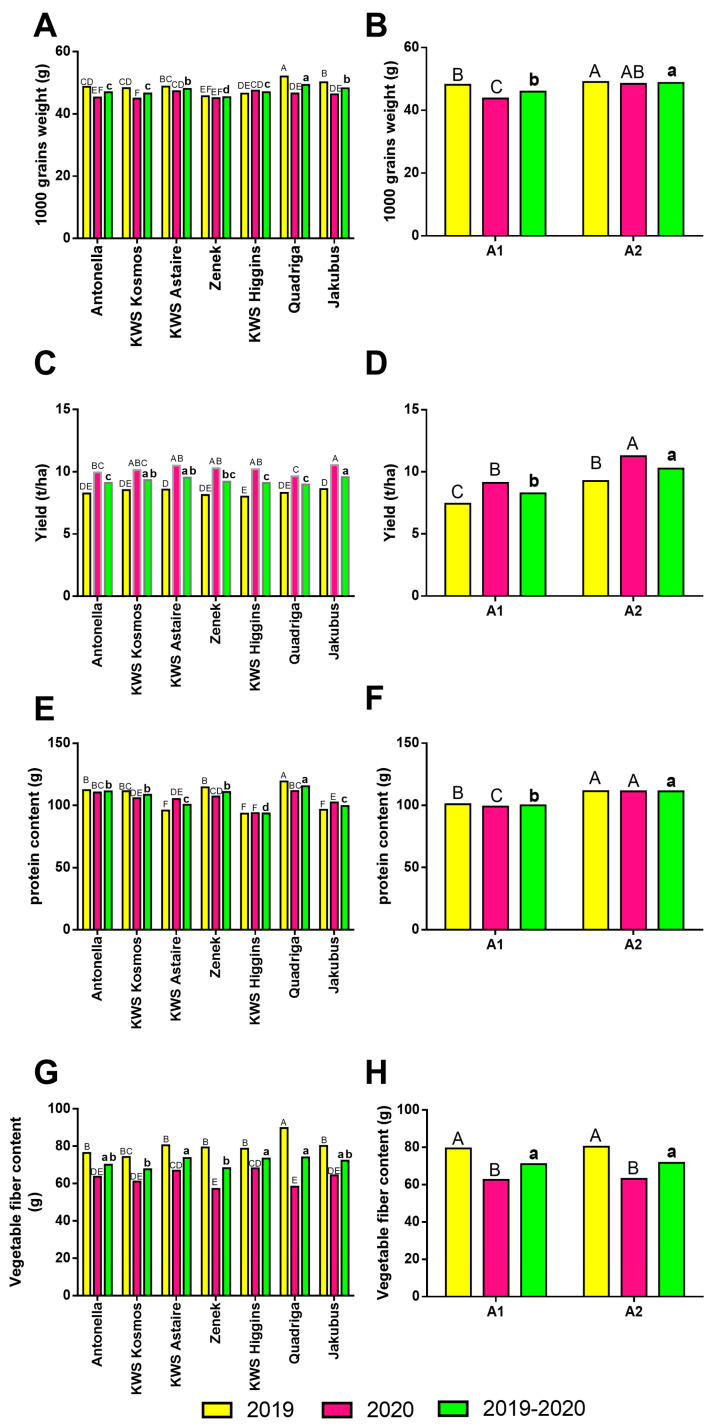
Effect of cultivar and agrotechnical level on some parameters of winter barley grain: (**A**,**B**): thousand-grain weight; (**C**,**D**): yield; (**E**,**F**): protein content; (**G**,**H**): vegetable fibre content. The same letters (A,B,C…; a,b,c…) separately indicate significance for the years 2019, 2020, and the mean from years 2019–2020 (*p*-0.01); A1—basic agrotechnical level; A2—intensive agrotechnical level.

Statistical analyses revealed that the examined winter barley cultivars differed significantly in terms of thousand-grain weight across the study years (Figure 1A). The highest mean thousand-grain weight was recorded for the cultivar Quadriga (49.31 g), while the lowest was observed for Zenek (45.38 g).

The agrotechnical level had a significant effect on this yield component (Figure 1B). It was demonstrated that the intensive agrotechnical level (A2) significantly increased the thousand-grain weight, with a statistically significant improvement observed in both years of the study compared with the basic agrotechnical level (A1).

Winter barley yield was dependent on the agrotechnical level (Figure 1D). Higher yields were obtained at the A2 level in both years of the study, averaging 11.26 t·ha^−1^ in 2020 and 9.26 t·ha^−1^ in 2019. At the A1 level, yields were significantly lower. In the first year of the study, the mean yield across cultivars was 7.43 t·ha^−1^, while in 2020 it was higher, reaching 9.10 t·ha^−1^. The study included an evaluation of the yield performance of seven winter barley cultivars during the 2019–2020 seasons (Figure 1C).

The analysis revealed that the highest yield was obtained for the cultivar Jakubus (9.57 t·ha^−1^), while KWS Astaire (9.34 t·ha^−1^) and KWS Kosmos (9.34 t·ha^−1^) produced slightly lower but not statistically different yields. Significantly lower yields were recorded for the cultivars Quadriga (mean 8.98 t·ha^−1^ across both years), Antonella (mean 9.10 t·ha^−1^), and KWS Higgins (mean 9.11 t·ha^−1^).

Statistical analysis also included an assessment of the effects of agrotechnical level and cultivar on the protein content of winter barley grain (Figure 1E). The highest protein content was recorded for the cultivar Quadriga (115.44 g), whereas the lowest was found in the cultivar KWS Higgins (93.56 g). The agrotechnical level influenced the protein content of barley grain in both years of the study. Application of the A2 level improved grain quality by increasing protein accumulation by 10.38% in the first year and 12.32% in 2020, resulting in mean protein contents of 111.44 g and 111.28 g, respectively (Figure 1F).

Analysis of the effects of the examined factors on fibre content showed that both cultivar and agrotechnical level influenced this parameter (Figure 1H). The cultivars Quadriga, KWS Higgins, and KWS Astaire exhibited the highest fibre content, while KWS Kosmos and Zenek had the lowest. However, when analysing the effect of agrotechnical level on fibre content, it was found that in both study years, the agrotechnical level did not significantly differentiate fibre content (Figure 1G).

Based on Spearman’s correlation analysis, certain relationships between the variables studied were identified. A relatively strong positive correlation was found between fibre content and thousand-grain weight (R = 0.51), and a negative correlation between yield and fibre content (R = −0.41). Furthermore, higher protein content was recorded in higher-yielding plots (R = 0.27) and in those with a greater thousand-grain weight (R = 0.33).

### 2.2. Mycotoxin Content

**Figure 2:**
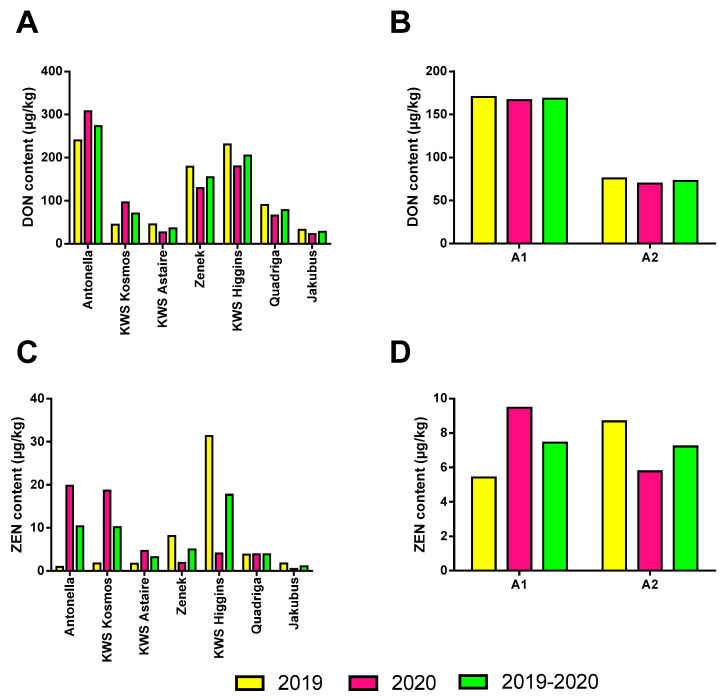
Effect of cultivar and agrotechnical level on some parameters of winter barley grain: (**A**,**B**): DON content; (**C**,**D**): ZEN content, A1—basic agrotechnical level; A2—intensive agrotechnical level.

Analysis of mycotoxin levels showed that the presence of DON in winter barley grain may depend on the agrotechnical level; however, these results require further confirmation (Figure 2B).

Higher DON concentrations were observed in the grain of various cultivars grown under the A1 level in both study years, averaging 170.36 µg·kg^−1^ in 2019 (41.2–377.7 µg·kg^−1^) and 166.61 µg·kg^−1^ in 2020 (27.7–490.8 µg·kg^−1^). The A2 agrotechnical level contributed to a reduction in DON accumulation, as lower values were recorded in both years (75.62 µg·kg^−1^ in 2019 and 69.69 µg·kg^−1^ in 2020).

The cultivars included in the experiment also differed in DON content in both years of the study (Figure 2A). Analysis indicated that the highest DON contamination occurred in the grain of cultivar Antonella (273.6 µg·kg^−1^), whereas the lowest levels were found in KWS Astaire (35.56 µg·kg^−1^) and Jakubus (27.40 µg·kg^−1^).

The qualitative analysis of grain in terms of mycotoxin content also included the determination of ZEN levels in winter barley grain (Figure 2C,D). The study revealed relatively low ZEN concentrations, ranging from 0.5 to 33.78 µg·kg^−1^. In the first year of the study, higher ZEN contamination was recorded under the A2 agrotechnical level (8.68 µg·kg^−1^), while grain produced under the A1 level showed a lower ZEN content (5.42 µg·kg^−1^). In the second year of the study, the opposite trend was observed: stronger ZEN contamination occurred under the A1 agrotechnical level, averaging 9.5 µg·kg^−1^, whereas under A2 the mean ZEN content was 5.8 µg·kg^−1^ (Figure 2D). The cultivars also differed in their levels of ZEN accumulation across the study years. The highest contamination was recorded in the grain of cultivar KWS Higgins (17.68 µg·kg^−1^), while the lowest was found in Jakubus (1.13 µg·kg^−1^) (Figure 2C). The new Regulation (EU) No. 915/2023 sets maximum levels for specific mycotoxins, including aflatoxins, ochratoxin A, patulin, deoxynivalenol, zearalenone and others. This regulation introduces significant updates, improves the clarity of provisions, and establishes new standards for monitoring these toxins in foodstuffs. The standard specifies precise thresholds for unprocessed cereal grains: 1250 µg·kg^−1^ for DON and 100 µg·kg^−1^ for ZEN. The winter barley grain tested in this study did not exceed the permissible limits for either DON or ZEN. However, it should be emphasised that the analyses were performed on the material immediately after harvest, so storage conditions did not affect mycotoxin levels.

Statistical analyses also included the assessment of the relationship between grain contamination with the mycotoxins DON and ZEN and the yield structure components, protein content, and fibre content (Table 2). Based on Spearman’s rank correlation analysis, higher DON concentrations were observed in lower-yielding plots (R = −0.35). In addition, DON content negatively affected the thousand-grain weight (R = −0.28). However, given the low Spearman correlation coefficients and the study’s design, the results obtained require confirmation in further research.

### 2.3. Mycological Analysis of Grain

Mycological analysis of grain from various winter barley cultivars grown under two agrotechnical levels during 2019–2020 resulted in the isolation of 1351 fungal isolates, including both filamentous and yeast-like fungi (Table 3).

The mycobiome of winter barley grain was dominated by *Alternaria alternata*, *Fusarium graminearum*, *Bipolaris sorokiniana*, and representatives of the genus *Penicillium*. Analysis of the effect of agrotechnical level on the species composition and abundance of fungi colonising winter barley grain showed a decrease in the number of isolates with increasing agrotechnical intensity. Specifically, a reduction of approximately 15.5% in the number of fungal cultures was observed under the A2 level. Individual cultivars responded differently to the agrotechnical treatments; notably, in the high-yielding cultivar Jakubus, grown under the A2 level, a 27.7% reduction in the number of isolated fungi was recorded following the application of the more intensive agrotechnical regime.

The mycological analysis also enabled the classification of the isolated fungi into the following trophic categories: [STP_F]—seed-transmitted pathogenic fungi; [RGQ_F]—fungi that reduce seed and grain quality; [S_F]—storage fungi; and [NDE_F]—fungi with no detrimental effects on grain (Table 3).

These classifications, determined according to cultivar and agrotechnical level, allowed for comparisons of biodiversity among the experimental variants in terms of the occurrence of the respective fungal categories (Table 4). Analysis of the biodiversity of the mycobiome with respect to individual fungal groups showed that the values of the Relative frequency index (Rf) depended on both cultivar and agrotechnical level. The highest Rf values were recorded for the group of fungi that reduce seed and grain quality ([RGQ_F], 2.68–6.70). The seed-transmitted pathogenic fungi group ([STP_F]) exhibited lower Rf values (0.36–2.68), whereas the lowest Rf values were found for the fungi that have no detrimental effects group ([NDE_F]) (Table 4). In the high-yielding cultivar Jakubus grown under the A2 level, the Relative frequency of seed-transmitted pathogenic fungi decreased from Rf = 2.5 (A1) to Rf = 1.25 (A2). At the same time, this cultivar showed an increase in the relative frequency of fungi that have no detrimental effects, from Rf = 0.36 (A1) to Rf = 1.43 (A2).

Analysis of the Dominance index (Y) confirmed the significant contribution of fungal communities responsible for the deterioration of winter barley grain quality ([RGQ_F], Y = 0.07–0.37) (Table 4).

The Species richness index (S) of the fungal communities indicated a predominant role of the STP_F and RGQ_F groups, while the remaining groups exhibited lower species richness.

Biodiversity assessment of the fungal communities colonising winter barley seeds also included the determination of the Margalef index (D′) and the Shannon–Wiener index (H′) (Table 4). The obtained values indicated a substantial contribution of pathogenic fungi and fungi that deteriorate grain quality, and a minor contribution of storage fungi within the mycobiome.

For the cultivar Jakubus, which exhibited the highest yield potential, an increase in the agrotechnical level resulted in a decrease in both D′ and H′ values for the STP_F group (D′A1 = 2.10 → D′A2 = 1.89); (H′A1 = 0.34 → H′A2 = 0.24) and the RGQ_F group (D′A1 = 2.18 → D′A2 = 1.66); (H′A1 = 0.61 → H′A2 = 0.47). At the same time, an increase in the biodiversity of NDE_F fungi was observed for this cultivar, expressed by higher values of both D′ (D′A1 = 0.72 → D′A2 = 1.08) and H′ (H′A1 = 0.07 → H′A2 = 0.26) (Table 4).

Analysis of the Dominance index (λ) demonstrated that the mycobiome of winter barley was dominated by species responsible for the deterioration of grain quality ([RGQ_F]) (Table 4).

Statistical analyses also included the assessment of the relationships between the biodiversity indices of the different fungal groups present in the winter barley grain mycobiome and yield, protein content, fibre content, and mycotoxin (DON and ZEN) concentrations, using Spearman’s rank correlation (Table 5).

Statistical analysis confirmed that winter barley yield was influenced by the diversity of seed-transmitted pathogenic fungi, as indicated by the Species richness (S) index (R = –0.20). This relationship suggests that greater species diversity among pathogenic fungi was associated with lower yields. Moreover, the occurrence of fungi that reduce seed and grain quality was correlated with barley yield, as evidenced by Spearman’s rank correlation coefficients obtained for the biodiversity indices Dominance (Y), Margalef index, and Shannon–Wiener index (Table 5). Grain quality, expressed in terms of protein content, was negatively affected by the Dominance (λ) of fungi that reduce grain quality, as confirmed by a correlation coefficient of R = −0.40, *p* = 0.01.

Fibre content was strongly influenced by the biodiversity of pathogenic species, showing a significant negative correlation with several diversity indices: Relative frequency, Dominance (Y), Species richness, Margalef index, Shannon–Wiener index, and Dominance index (λ) (Table 5).

The thousand-grain weight was shaped by the diversity of both pathogenic fungi and those reducing grain quality, commonly referred to as field fungi. Higher thousand-grain weights were recorded when the Relative frequency and Shannon–Wiener index values of storage fungi were higher, and when fungi that have no detrimental effects on grain predominated in the fungal community (Table 5).

The occurrence of DON in winter barley grain was influenced by the biodiversity indices of fungi that reduce grain quality, storage fungi, and pathogenic fungi. Additionally, ZEN contamination was higher when the Relative frequency, Dominance (Y), and Margalef index values in the mycobiome increased (Table 5).

## 3. Discussion

### 3.1. Yield, Genotypes, and the Effect of Agrotechnical Practices

Winter barley is primarily cultivated for feed production, which makes it a crop focused on achieving high grain yield together with elevated protein and dietary fibre content. To fully exploit the genetic potential of winter barley cultivars, it is essential to provide the plants with optimal growing conditions throughout the vegetation period. Among the various factors affecting yield, agrotechnical management plays a decisive role, and fertilisation is the most influential element determining both the quantity and quality of the grain. Adequate nitrogen nutrition enhances grain yield and protein accumulation; however, excessive nitrogen application can result in excessive vegetative growth and increased disease incidence, ultimately reducing both yield and grain quality [30]. Modern cultivars exhibit improved performance and a stronger response to fertilisation compared with older cultivars, which highlights the important role of breeding progress in shaping barley productivity [31]. The simplest approach to achieving high yields in feed barley cultivation is the application of higher nitrogen doses, as demonstrated by both national and international studies [32,33]. Increased fertilisation promotes higher thousand-grain weight and grain yield, which was also confirmed in our study. Intensive agrotechnical management, involving higher nitrogen application, significantly increased thousand-grain weight in both years of the experiment. Other studies indicate that optimal fertilisation improves yield mainly by increasing the number of grains per square metre, but not necessarily the thousand-grain weight [34]. Among the examined cultivars, Quadriga produced the highest mean thousand-grain weight across both years. It is noteworthy that all cultivars used in the experiment were six-row types. In this study, the intensive level of agrotechnology included a package of treatments consisting of high levels of nitrogen fertilisation, fungicides, growth regulators and microelements, which was compared to the low agrotechnology level. Such a combination of treatments, as proposed in our scheme, is practised by farmers; therefore, on the one hand, the use of such an experimental design is justified due to the practical implications of the research results. On the other hand, it causes difficulties in interpreting the component results, e.g., assessing the direct impact of individual elements of the treatment package, such as fertilisation or the use of protection, on the research results obtained in this study.

Winter barley yield was also significantly higher under the intensive agrotechnical level, and this effect was consistent across both study years. Among the tested genotypes, Jakubus achieved the highest yield (9.57 t·ha^−1^), followed closely (without statistically significant differences) by KWS Astaire (9.34 t·ha^−1^) and KWS Kosmos (9.34 t·ha^−1^). The influence of agrotechnical level and cultivar on protein content in the harvested grain was also examined. The intensive agrotechnical system, which included increased fertilisation, promoted greater protein accumulation in both years of the study. The highest protein content was found in the grain of the cultivar Quadriga (115.44 g). Given the feed-related importance of winter barley, the fibre content of the grain was also assessed. The applied agrotechnical treatments did not significantly affect fibre content in either year of the study. However, analysis of the cultivar factor showed that Quadriga, KWS Higgins, and KWS Astaire had significantly higher fibre content compared with the other cultivars.

The obtained results—particularly those related to grain quality—indicate a high production potential for Quadriga. However, its yield data suggest that, apart from thousand-grain weight, other yield components require further optimisation to achieve a synergistic improvement in total grain yield for this cultivar.

Overall, the present study clearly demonstrates that cultivating winter barley under intensive agrotechnical conditions, involving higher nitrogen fertilisation, the application of multi-micronutrient foliar fertiliser, growth regulators, and fungicides, positively affected both the production potential of the plants and the quality of the harvested grain. According to Kazimierczak et al. (2025) [33], an integrated agronomic approach combining the selection of optimal genotypes with appropriate cultivation practices can ensure high yields with desirable quality parameters.

### 3.2. Occurrence of Mycotoxins, Fungi, and Correlations

Our study presents a new perspective on the occurrence of plant diseases by challenging the traditional “one pathogen–one disease” paradigm, as disease manifestation results from the coexistence of multiple interacting factors that shape fungal communities determining plant health or disease [35]. Heterogeneous microbial communities colonising plant organs interact through physical and chemical mechanisms [36]. Thus, the plant microbiota should be viewed as a complex system of microorganisms functioning in mutual interaction. It includes soterobionts, which act as a protective layer providing a barrier against pathogen invasion [37]. Other microorganisms within this network may serve as potential pathogens, and an increase in their abundance may lead to disease development or symptom intensification [38]. Some members of this microbial consortium may coexist with strong pathogens in synergistic relationships [35]. Microbial consortia associated with plant disease pathogenesis form dynamic interactions from the infection stage through subsequent phases of disease development, accompanying infection progression—these complex networks are now defined as the pathobiome [39]. Similarly, the seed microbiome plays a key role in seedling health by supplying nutrients and enhancing resistance to abiotic and biotic stresses [37]. It should be emphasised that the seed microbiome can be vertically transmitted, suggesting that interactions among microorganisms colonising seeds are essential for maintaining plant health, both during early developmental stages [40] and throughout seed storage. Seeds, therefore, represent both the beginning and the end of the plant life cycle [41]. While the contribution of the seed mycobiome composition to seedling health is well established [40], quantitative and qualitative changes occur during seed storage. Under suboptimal temperature and humidity conditions, these changes may lead to the dominance of fungal species responsible for mycotoxin accumulation and deterioration of seed quality [42]. Guided by this understanding, the present study classified fungal species into functional groups based on their impact on seed material and analysed their relationships with yield, thousand-grain weight, protein and fibre content in the grain. Our findings based on the mycological analysis demonstrated that fungi belonging to the group [RGQ_F]—fungi that reduce seed and grain quality were the most abundant, as confirmed by biodiversity indices (Rf, Y, S, H′, and λ). While seeds in early developmental stages may engage in adaptive interactions with microorganisms to support development [40], post-harvest conditions—such as temperature, humidity, and storage hygiene (e.g., silo cleanliness)—influence the dynamics of seed-associated microbiota. These factors may promote or suppress certain fungal groups, ultimately determining the suitability of seed material for further use.

The grain analysed in this study originated directly from the field, and the results clearly confirmed the dominance of fungi negatively affecting seed quality, as well as seed-transmitted pathogens, both classified as field fungi. Przybylska-Balcerek et al. (2020) [43] found that barley grain mycobiota were dominated by *Aspergillus*, *Penicillium*, and *Fusarium* species, as well as *Mucor*, *Alternaria*, and *Cladosporium*, which is consistent with our findings. However, in our samples, storage fungi were relatively rare, whereas *Fusarium* species—particularly *F. graminearum*—were frequently isolated. Consequently, both DON and ZEN were detected in the grain.

According to Kazimierczak et al. (2025) [33], DON is the most common mycotoxin found in winter barley grain in Poland, with concentrations ranging from 84 to 289 µg·kg^−1^, depending on cultivar and fertilisation level. DON accumulates as a result of Fusarium head blight (FHB) infection, which develops during cereal flowering and can persist until harvest under favourable weather conditions, such as high humidity [32]. Przybylska-Balcerek et al. (2020) [43], analysing 44 barley samples collected during 2015–2016, reported relatively low DON concentrations, averaging 41.0 and 52.0 µg·kg^−1^, respectively.

Similarly, in the present study, the concentrations of both mycotoxins did not exceed permissible EU levels, although contamination levels of DON and ZEN were higher than those reported by Przybylska-Balcerek et al. (2020) [43]. Mycotoxin contamination was influenced by both agrotechnical level and cultivar. Differences in DON and ZEN accumulation were observed among cultivars: the highest levels were detected in Antonella (273.63 µg·kg^−1^ DON; 10.36 µg·kg^−1^ ZEN), whereas the lowest occurred in Jakubus (27.40 µg·kg^−1^ DON; 1.13 µg·kg^−1^ ZEN).

Based on our findings, barley grain contamination with mycotoxins was probably associated with the presence of *F. culmorum* and *F. graminearum*, both known producers of these toxins in cereal grains in Poland [9]. Both species were frequently isolated from all grain samples, with *F. graminearum* clearly dominating—a finding consistent with other studies identifying it as the primary FHB pathogen in Poland [11,44,45,46].

Previous research has shown that FHB influences the protein content of grain [47]. In our study, a negative correlation was found between protein content and the Dominance index (λ) of the fungal community classified as fungi that reduce grain quality (RGQ_F) (R = –0.40; *p* = 0.01). Moreover, analysis of the relationship between fungal diversity and yield showed that yield reduction was associated with a higher Species richness (S) of seed-transmitted pathogenic fungi (R = –0.20; *p* = 0.05*), as well as with Dominance (Y) (R = –0.21; *p* = 0.05*), and biodiversity indices Margalef (D′) (R = –0.26; *p* = 0.05*) and Shannon–Wiener (H′) (R = –0.32; *p* = 0.05*) for the group of fungi that reduce seed and grain quality.

*Fusarium* species, which in the present study constituted a substantial proportion of the fungi responsible for the deterioration of grain quality, are known to synthesise a wide range of secondary metabolites with diverse biological properties, including antibacterial, antifungal, phytotoxic, anti-allergic, and cytotoxic effects [48]. Secondary metabolites produced by *F. graminearum* include kaneoheoic acids G and H [49], fusarins X_1_ and Y [50], fusahexin [51], fusaranes A and B [52], and gramipiperazines A and B [53], all of which possess primarily antibacterial and cytotoxic properties.

It should be emphasised that such studies are ongoing and may provide additional insights, as different strains of the same species can vary in their metabolic capabilities. The dominance of specific fungal taxa on seeds is determined not only by storage conditions but also by evolutionary adaptations that confer particular metabolic traits. Fungi colonising grain therefore form a complementary consortium that remains in a state of dynamic equilibrium. Understanding the network of interspecific relationships among fungal taxa could support the development of more effective seed storage strategies. Currently, most storage practices focus primarily on mitigating the adverse effects of mycotoxins. A new perspective that considers the ecological roles of coexisting fungal groups as an integrated seed mycobiome warrants further in-depth investigation.

## 4. Conclusions

Based on the conducted field experiments, it can be concluded that the intensification of winter barley agrotechnical practices—including higher nitrogen fertilisation, the application of multi-micronutrient foliar fertilisers, growth regulators, and fungicides—had a positive effect on both plant yield and the feed quality of the harvested grain, particularly in terms of protein and fibre content. The cultivars differed in their response to increased fertilisation and the application of additional crop protection treatments, indicating the necessity for careful selection of cultivars suited to specific site and management conditions by agricultural producers.

Analysis of the impact of agrotechnology on the species composition and abundance of fungi colonising winter barley grain demonstrated a decrease in fungal isolates with increased agrotechnological input. This reduction was accompanied by lower levels of DON and ZEN mycotoxins. Mycological assessments and Spearman rank correlation analyses between fungal biodiversity indices and yield components indicated that the grain mycobiome was dominated by field fungi species, particularly *Fusarium*, which are responsible for infection and contribute to seed quality deterioration. High dominance index [λ] values for these fungi were associated with decreased grain protein content. Yield reduction correlated with species richness (S), the presence of seed-transmitted pathogenic fungi, dominance (Y), Margalef’s index (D’), and Shannon–Wiener index (H’) of the fungal community, all of which negatively affected seed and grain quality. Elevated biodiversity indicators for seed-transmitted pathogenic fungi were linked to decreased vegetable fibre content and lower 1000-grain weight.

Comprehensive understanding of the interrelationships among fungal groups and their influence on crop yield and structure, in conjunction with production factors, necessitates further research to optimise resource use, which is critical for sustainable agriculture and for better quality of agro-products. It should be emphasised that an intensive level of agrotechnology constitutes a package of measures which, when implemented together, improves both the yield and quality of barley grain in various ways, including reducing the occurrence of *Fusarium* fungi and the mycotoxins DON and ZEN. Such an agrotechnical strategy should be adopted by producers to achieve both economic benefits and to ensure feed and food safety. Based on the results obtained, it can be concluded that the grain mycobiome develops during the growing season, and the level of agrotechnology determines not only the quality parameters of the grain but also the occurrence of specific groups of fungi and mycotoxins. In our view, research should continue to explain this phenomenon thoroughly, taking into account field experiments involving multi-level analysis, in which the impact of specific agrotechnical treatments (nitrogen fertilisation, fungicide protection, and the use of micronutrients) will be assessed in comparison with a package of treatments that offers an attractive solution for agricultural practice regarding the composition of the grain mycobiome and the accumulation of mycotoxins.

## 5. Materials and Methods

### 5.1. Field Experiment

The present study was based on field experiments conducted during the 2018/2019 and 2019/2020 growing seasons at the experimental fields (51°11′ N, 17°51 E) of the Institute of Agroecology and Plant Production, Wrocław University of Environmental and Life Sciences (Poland).

Weather conditions during both growing seasons are presented in Tables S1 and S2. In the 2018/2019 season, the average monthly temperature was higher than the long-term average for most months, except January, May, and July. Overall, it can be said that the thermal conditions were favourable for the development and growth of winter barley. The total rainfall during the autumn growing season (September–November) was 115.2 mm, which significantly exceeded the optimal amount for barley. Low rainfall in June and July (27.0 mm and 44.5 mm, respectively) and high average temperatures (22.1 °C and 19.3 °C) enabled the grain in the ears to dry properly and accelerated harvesting (Table S1). In 2019/2020, average monthly temperatures were higher compared to the long-term (1986–2015) average, except in May. Noteworthy is the high amount of rainfall in May and June, as well as rainfall in early July. However, this did not affect the drying of the grain, and the harvest was carried out on time (Table S2).

The experiments were established as two-factor trials (two plots for the experimental variant) in a split-block design. The first factor was the agrotechnical level, and the second was the winter barley cultivar.

Agrotechnical levels:

A1—basic agrotechnical level: nitrogen fertilisation of 60 kg·ha^−1^, applied in two doses: 50 kg·ha^−1^ N at the onset of spring vegetation and 10 kg·ha^−1^ N at the beginning of stem elongation.

A2—intensive agrotechnical level: nitrogen fertilisation of 100 kg·ha^−1^, applied in two doses: 60 kg·ha^−1^ N at the onset of spring vegetation and 40 kg·ha^−1^ N at the beginning of stem elongation; two fungicide applications; application of a growth regulator and a multi-micronutrient foliar fertiliser. Intensive agricultural technology integrates various methods used in agriculture to achieve high yields with favourable quality parameters. This approach aligns with the most commonly used strategy in agriculture and has been compared to the basic variant, which involves only low-dose nitrogen fertilisation. Detailed information on cultivation, such as the fertilisers and substances used and the timing of their application (dates and growth stages of winter barley), are presented in Table S3 for the 2018/2019 season and in Table S4 for the 2019/2020 season. All the substances (products) used were legally allowed for application in winter barley cultivation during the years of the field experiment.

Winter barley cultivars: Antonella, KWS Kosmos, KWS Astaire, Zenek, KWS Higgins, Quadriga, and Jakubus.

In both study years, the preceding crop for winter barley was winter oilseed rape. Sowing was performed on 1 October 2018 and 27 September 2019 using a plot seeder at a depth of 3 cm and a row spacing of 12.5 cm. Each plot measured 15 m^2^ (10 × 1.5 m). Harvest took place on 1 July 2019 and 10 July 2020 using a small-plot combine harvester. Immediately after harvest, the grain yield from each plot was determined and converted to yield per hectare, assuming 13% grain moisture. The thousand-kernel weight (Weight of 1000 grains) was also determined directly after harvest. The harvested grain was cleaned using a MiniBatt grain cleaning machine (Godé, Le Catelet, France) and subsequently subjected to chemical and mycological analyses. Grain obtained from the 2018/2019 growing season is hereafter referred to as “2019,” while grain from the 2019/2020 season is designated as “2020.”

### 5.2. Chemical Analyses of Grain

To determine the chemical composition of the grain, 1 kg samples were collected from each plot after harvest. The quantity of material used for each analysis depended directly on the specific laboratory methodology applied. The analyses included the determination of crude protein and crude fibre contents using the following methods:

Total protein content: modified Kjeldahl’s method, involving mineralization of nitrogen compounds in sulfuric acid (98% pure, analytical grade) at a temperature of approx. 400 °C, followed by distillation and titration using a Büchi Distillation Unit K-355 (BUCHI Labortechnik AG, Uster, Switzerland). When converting total nitrogen to total protein, the coefficient 5.7 was used.

Vegetable fibre content: Henneberg–Stohmann’s method, using a Velp extraction apparatus (VELP Scientifica Srl, Usmate, Italy). This method involves the successive hot extraction of a plant material sample with a diluted solution of sulfuric acid (98% pure, analytical grade) and potassium hydroxide (98% pure, analytical grade), followed by an organic solvent (distilled water).

Both the acid and the lye were manufactured by Stanlab Sp. z o. o. (Lublin, Poland).

### 5.3. Mycological Analysis of Winter Barley Grain

Mycological analyses were conducted on grain collected from each plot. Toxicological analyses were performed to determine mycotoxin content, creating a bulk sample for each research variant, each totalling 4 kg of grain. These prepared samples were then delivered to the laboratory for analysis. The species composition of fungi colonising the grain was examined immediately after harvest in each year of the study. From each sample, one hundred grains were randomly selected and rinsed three times with distilled water for 30 s each. They were then treated with 70% ethanol for 5 min, followed by a rinse with a 1% sodium hypochlorite solution (NaOCl, 6–14% active chlorine, EMPLURA^®^, catalog no. 1.05614, Sigma-Aldrich, Darmstadt, Germany) for another 5 min, and finished with three additional rinses with sterile distilled water. The cleaned grains were placed in Petri dishes on potato dextrose agar (PDA medium, which contained: agar, catalog no. A1296, microbiologically pure; glucose, catalog no. G7021, ≥99.5%, both Sigma-Aldrich, Darmstadt, Germany), with seven grains per dish, and incubated under laboratory conditions at a temperature of 20–22 °C for 7–14 days. After this incubation, fragments of the mycelia were transferred to new sterile Petri dishes containing fresh PDA medium. Following an additional incubation of 14–20 days, the resulting fungal colonies were identified to the species or genus level based on morphological characteristics, including mycelium type, pigmentation, shape, and size, as well as the formation of mycelia and the presence of spore forms. If obtaining spores proved challenging, the fungal cultures were subjected to UV radiation in a sequence of 12 h of UV exposure followed by 12 h of darkness in a thermostat (Gallenkamp Economy incubator, Leicester, UK). After another 14 days, the cultivated fungi were pre-selected based on their morphological traits. A light microscope (Olympus CX40, Tokyo, Japan) was used for microscopic examination to identify the fungi, utilising the available identification keys [54,55,56,57].

### 5.4. Data Analysis of the Mycobiome of Winter Barley Grain

The following definitions were used to describe the trophic groups present in the mycobiome of winter barley grain: [STP_F]—seed-transmitted pathogenic fungi; [RGQ_F]—fungi that reduce seed and grain quality; [S_F]—storage fungi; [NDE_F]—fungi that have no detrimental effects on grain [45]. The biodiversity indices (Table 6) were used to quantify the relationships between abundance, distribution preference, and fungal community composition, taking into account individual fungal groups that influence the quality of winter barley grain according to the above categorisation.

### 5.5. Quantification of Mycotoxin Contamination

For the DON and ZEN analyses, grain from the field plots belonging to a specific cultivar (two plots) × agrotechnical level × year combination was combined into a single composite bulk sample prior to laboratory quantification. The experimental unit for mycotoxin determination was the composite sample, from which three analytical replicates were taken. Grain samples (100 g each) were ground into a fine powder and homogenised. For each sample, three analytical replicates (25 g each) were weighed into 250 mL conical flasks. Acetonitrile, gradient grade for liquid chromatography (LiChrosolv^®^ Merck, Darmstadt, Germany) ≥99.9%, catalog no. 1.00030), methanol, gradient grade for liquid chromatography (LiChrosolv^®^, ≥99.9%, catalog no. 1.06007), and water for liquid chromatography (LiChrosolv^®^, HPLC grade, catalog no. 1.15333) were sourced from Merck (Darmstadt, Germany) and used throughout the study. Deoxynivalenol (DON) analytical standard (≥98%, certified reference material) and zearalenone (ZEN) analytical standard (≥98%, certified reference material) were obtained from Merck (Sigma-Aldrich, Darmstadt, Germany). DON was extracted with 100 mL of water for 60 min using a mechanical shaker (Innova 2100, New Brunswick Scientific, Edison, NJ, USA) and then filtered. ZEN was extracted with 100 mL of acetonitrile:water (90:10, *v*/*v*) for 60 min using a mechanical shaker (Innova 2100, New Brunswick Scientific, Edison, NJ, USA) and filtered; prior to immunoaffinity clean-up, the ZEN extracts were diluted 1:10 (*v*/*v*) with water. Extracts were purified using immunoaffinity columns (DON-Test™ and Zearala-Test™, VICAM, Watertown, MA, USA) according to the manufacturer’s recommendations. The columns were washed with 5 mL of water, and mycotoxins were eluted with 1.5 mL of methanol. The eluates were evaporated under a stream of nitrogen (technical grade 3.0, ≥99.9%; Messer Polska sp. z o.o., Chorzów, Poland) and reconstituted in 0.5 mL of the corresponding mobile phase. HPLC analyses were performed on a Shimadzu LC-20AD system equipped with UV and fluorescence detectors, using a Jupiter 5 µm C18 300 Å column (250 × 4.6 mm; Phenomenex, Torrance, CA, USA) at 24 °C with an injection volume of 100 µL. DON was determined by UV detection at 220 nm using an isocratic mobile phase of water:acetonitrile (90:10, *v*/*v*) at a flow rate of 1.0 mL min^−1^. ZEN was determined by fluorescence detection (λ_ex = 274 nm, λ_em = 440 nm) using an isocratic mobile phase of acetonitrile:water:methanol (46:46:8, *v*/*v*/*v*) at a flow rate of 0.5 mL min^−1^. Quantification was performed by external calibration. Primary stock standard solutions of DON and ZEN were prepared in-house from certified reference materials. Working calibration stock solutions were prepared by appropriate dilution of the primary stock solutions. Separate six-point calibration curves were used for DON and ZEN. For DON, the calibration range was 156.25–5000 ng mL^−1^. For ZEN, the calibration range was 0.25–20.0 ng mL^−1^. The limits of detection (LOD) and quantification (LOQ), estimated using the signal-to-noise approach (S/N = 3 and 10, respectively), were 5 and 10 µg kg^−1^ for DON and 0.15 and 0.50 µg kg^−1^ for ZEN, respectively.

### 5.6. Statistical Analysis

The statistical calculations were performed by means of three-way analysis of variance using the Tukey test (HSD), and values of *p* < 0.01 were considered significant. STATISTICA 13.3 software (TIBCO Software Inc., Palo Alto, CA, USA, 2017 was used for the mathematical analyses.

To determine the relationship between yield and elements shaping the yield, protein content, as well as biodiversity indices characterising the composition of the mycobiome–taking into account individual categories of fungi influencing the quality of winter barley grain—Spearman’s rank correlations (*—significant at *p* ≤ 0.05; **—significant at *p* ≤ 0.01) were determined.

## Figures and Tables

**Table 1:** The effects of the main factors (barley cultivar [C], agrotechnical level [AL] and year [Y]) and their interactions on the selected barley parameters determined by three-way ANOVA.

Parameter	Cultivar (C)	Agrotechnical Level (AL)	Year (Y)	C × AL	C ×Y	AL × Y	C × AL × Y
Yield	**	**	**	**	**	**	**
Protein content	**	**	*	**	**	**	**
Vegetable fibre content	**	ns	**	**	**	**	**
Weight of 1000 grains	**	**	**	**	**	**	**

* Significant at *p* ≤ 0.05; ** significant at *p* ≤ 0.01; and ns—not significant.

**Table 2:** Spearman’s rank correlations between yield and some other parameters and mycotoxin content in winter barley grains.

Parameter	Protein Content	Vegetable Fibre Content	Weight of 1000 Grains	DON Content	ZEN Content
Yield	0.27 *	−0.41 **	0.17	−0.35	−0.15
Protein content		0.08	0.33 *	0.14	0.03
Vegetable fibre content			0.51 **	0.11	0.05
Weight of 1000 grains				−0.28	−0.22

*—significant at *p* ≤ 0.05; **—significant at *p* ≤ 0.01.

**Table 3:** Fungi isolated from winter barley grain depending on the cultivar and level of agrotechnology in the years of research 2019–2020.

Species/Genus of Fungus	Category of Fungi	Basic Agrotechnical Level (A1)							Intensive Agrotechnical Level (A2)							Total
		Antonella	KWS Kosmos	KWS Astaire	Zenek	KWS Higgins	Quadriga	Jakubus	Antonella	KWS Kosmos	KWS Astaire	Zenek	KWS Higgins	Quadriga	Jakubus	
*Acremonium*	NDE_F	1	1	4	4	6	1	2	0	3	3	0	0	0	2	27
*Alternaria alternata*	RGQ_F	41	45	27	47	38	52	31	27	38	39	48	12	35	34	514
*Areubasidium*	NDE_F	0	0	0	0	1	0	0	0	0	0	0	0	1	0	2
*Ascochyta*	STP_F	0	0	0	0	0	0	0	0	0	0	0	4	4	0	8
*Aspergillus*	S_F	0	0	1	3	0	0	1	0	0	2	0	0	1	0	8
*Bipolaris sorokiniana*	RGQ_F	0	3	10	6	5	2	13	1	3	1	12	12	9	1	78
*Botrytis cinerea*	STP_F	1	0	1	2	0	1	2	0	0	1	0	0	0	0	8
*Chaetomium*	STP_F	0	1	0	0	0	0	0	0	0	0	1	0	1	0	3
*Cladosporium cladospirioides*	RGQ_F	0	1	5	6	1	3	9	1	1	1	5	5	3	2	43
*Epicoccum*	RGQ_F	2	0	0	4	2	7	1	2	1	3	4	0	4	5	35
*Fusarium avenaceum*	STP_F/RGQ_F	1	2	1	1	0	0	2	3	1	0	3	0	0	1	15
*F. culmorum*	STP_F/RGQ_F	2	0	2	4	2	2	2	6	2	5	2	4	2	2	37
*F. graminearum*	STP_F/RGQ_F	25	11	13	22	28	11	14	18	15	6	6	7	15	8	199
*F. oxysporum*	STP_F/RGQ_F	0	0	0	0	1	0	1	0	0	0	1	1	1	0	5
*F. poae*	STP_F/RGQ_F	7	0	0	0	0	2	2	8		1	0	0	1	1	22
*F. tricinctum*	STP_F/RGQ_F	1	5	0	2	1	2	4	4	1	1	0	3	8	1	33
*Fusarium*	STP_F/RGQ_F	3	0	0	2	1	1	3	1	1	1	2	2	7	7	31
*Mucor hiemalis*	S_F	0	1	0	2	2	0	1	0	3	1	0	3	1	0	14
*Papularia*	NDE_F	1	3	0	1	0	0	0	4	2	1	0	7	0	3	22
*Penicillium* spp.	S_F	5	20	13	20	0	8	9	1	0	8	3	3	7	1	98
*Penicillium citrinum*	S_F	0	0	0	1	0	0	0	0	0	0	0	0	0	0	1
*Phoma glomerata*	STP_F	2	1	1	1	0	6	8	2	0	1	1	0	0	2	25
*Phytium*	STP_F	0	0	0	0	0	0	0	2	0	0	0	0	0	1	3
*Rhizoctonia*	STP_F	0	0	1	1	1	0	0	1	0	0	0	0	0	0	4
*Rhizopus nigricans*	STP_F	1	0	2	0	1	8	8	2	11	5	0	7	4	2	51
*Sclerotinia sclerotiorum*	STP_F	0	0	1	0	0	0	0	0	0	0	0	1	0	0	2
*Torula*	NDE_F	0	0	0	0	1	0	0	0	0	0	0	1	0	0	2
*Trichotecium roseum*	RGQ_F	0	0	1	0	2	1	0	0	0	3	1	0	0	0	8
Yeast-derived fungi	NDE_F	0	0	0	1	0	0	0	0	0	1	0	0	0	7	9
Non-sporulating fungi	NDE_F	5	0	3	9	3	5	2	1	3	2	1	2	4	4	44
Sum		98	94	86	139	96	112	115	84	85	86	90	74	108	84	1351

[STP_F]—seed-transmitted pathogenic fungi; [RGQ_F]—fungi that reduce seed and grain quality; [S_F]—storage fungi; [NDE_F]—fungi that have no detrimental effects on grain.

**Table 4:** The diversity of different categories of fungi, including seed-transmitted pathogenic fungi [STP_F], fungi that reduce seed and grain quality [RGQ_F], storage fungi [S_F] and fungi that have no detrimental effects on grain [NDE_F], in winter barley grain depending on the cultivar and level of agrotechnology in the years of research 2019–2020.

Diversity Index	Category of Fungi	Basic Agrotechnical Level (A1)							Intensive Agrotechnical Level (A2)						
		Antonella	KWS Kosmos	KWS Astaire	Zenek	KWS Higgins	Quadriga	Jakubus	Antonella	KWS Kosmos	KWS Astaire	Zenek	KWS Higgins	Quadriga	Jakubus
Relative frequency [Rf]	STP_F	1.52	1.16	0.63	0.98	0.45	1.34	2.50	2.32	0.36	0.54	0.63	1.25	2.68	1.25
	RGQ_F	5.00	5.71	3.84	5.98	4.55	6.70	5.54	5.18	5.27	4.73	5.98	2.68	5.89	6.07
	NDE_F	0.63	0.36	0.63	1.34	0.98	0.54	0.36	0.45	0.71	0.63	0.18	0.89	0.45	1.43
	S_F	0.54	1.96	1.43	2.32	0.27	1.43	1.70	0.27	1.25	1.43	0.36	1.16	1.25	0.27
Dominance [Y]	STP_F	0.02	0.01	0.00	0.01	0.00	0.02	0.06	0.05	0.00	0.00	0.00	0.02	0.07	0.02
	RGQ_F	0.25	0.33	0.15	0.36	0.21	0.45	0.31	0.27	0.28	0.22	0.36	0.07	0.35	0.37
	NDE_F	0.00	0.00	0.00	0.02	0.01	0.00	0.00	0.00	0.01	0.00	0.00	0.01	0.00	0.02
	S_F	0.00	0.04	0.02	0.05	0.00	0.02	0.03	0.00	0.02	0.02	0.00	0.01	0.02	0.00
Species richness [S]	STP_F	7	3	6	6	4	6	8	8	3	5	4	5	6	6
	RGQ_F	8	6	7	8	9	10	11	10	8	9	9	7	10	9
	NDE_F	3	2	2	4	4	2	2	2	3	4	1	3	2	4
	S_F	1	3	3	4	2	2	4	1	2	4	2	3	5	1
Margalef index [D’]	STP_F	2.12	0.78	2.57	2.09	1.86	1.85	2.10	2.15	1.44	2.23	1.54	1.52	1.47	1.89
	RGQ_F	1.49	0.96	1.33	1.43	1.78	1.85	2.18	1.97	1.47	1.76	1.66	1.47	1.91	1.66
	NDE_F	1.03	0.72	0.51	1.11	1.25	0.56	0.72	0.62	0.96	1.54	1.44	0.87	0.62	1.08
	S_F	0.56	0.65	0.72	0.92	0.91	0.36	1.02	0.91	0.38	1.08	0.72	0.78	1.52	0.91
Shannon–Wiener index [H’]	STP_F	0.30	0.14	0.17	0.15	0.11	0.20	0.34	0.44	0.08	0.13	0.03	0.24	0.30	0.24
	RGQ_F	0.42	0.33	0.45	0.43	0.43	0.44	0.61	0.58	0.34	0.38	0.18	0.50	0.61	0.47
	NDE_F	0.13	0.08	0.13	0.18	0.21	0.09	0.07	0.11	0.16	0.15	0.01	0.18	0.08	0.26
	S_F	0.11	0.05	0.06	0.12	0.12	0.07	0.03	0.11	0.10	0.09	0.01	0.18	0.08	0.22
Dominance index [λ]	STP_F	0.01	0.01	0.01	0.01	0.01	0.01	0.01	0.03	0.00	0.00	0.01	0.01	0.02	0.01
	RGQ_F	0.34	0.30	0.16	0.18	0.34	0.28	0.12	0.22	0.32	0.27	0.01	0.08	0.17	0.23
	NDE_F	0.01	0.01	0.01	0.01	0.01	0.01	0.01	0.01	0.01	0.01	0.01	0.01	0.00	0.01
	S_F	0.01	0.01	0.01	0.01	0.01	0.01	0.01	0.01	0.01	0.01	0.01	0.01	0.00	0.01

**Table 5:** Spearman’s rank correlation coefficients (R) between selected diversity indices of fungal groups isolated from grain and some indices of winter barley grain.

Parameter	Relative Frequency [Rf]				Dominance [Y]				Species Richness [S]				Margalef Index [D’]				Shannon–Wiener Index [H’]				Dominance Index [λ]			
	STP_F	RGQ_F	NDE_F	S_F	STP_F	RGQ_F	NDE_F	S_F	STP_F	RGQ_F	NDE_F	S_F	STP_F	RGQ_F	NDE_F	S_F	STP_F	RGQ_F	NDE_F	S_F	STP_F	RGQ_F	NDE_F	S_F
Yield	0.05	−0.12	−0.10	−0.28 *	0.08	−0.21 *	−0.10	−0.29 *	−0.20 *	−0.08	−0.05	−0.13	−0.07	−0.26 *	−0.07	−0.02	−0.06	−0.32 *	−0.05	0.00	0.03	0.11	0.02	−0.02
Protein content	0.14	−0.09	−0.13	0.03	0.14	−0.09	−0.16	0.20	0.19	−0.18	−0.20 *	0.18	0.10	−0.15	0.15	0.16	−0.19	0.05	0.18	0.17	0.05	−0.40 **	0.07	0.08
Vegetable fibre content	-0.57 **	−0.15	0.11	0.16	−0.57 **	−0.15	0.30 *	0.16	−0.48 **	0.17	0.26 *	0.12	−0.42 **	−0.26 *	0.12	−0.08	−0.61 **	−0.60 **	0.31 *	0.11	−0.63 **	−0.11	0.70 **	0.07
Weight of 1000 grains	−0.38 *	−0.09	0.08	0.39 *	−0.38 *	−0.09	0.08	0.39 *	−0.28 *	0.05	0.04	0.26 *	−0.16	−0.22 *	0.05	0.12	−0.41 **	−0.40 **	0.11	0.32 *	−0.48 **	−0.19	0.33 *	0.05

R **—significant at *p* = 0.01, R *—significant at *p* = 0.05, R—not significant.

**Table 6:** Biodiversity indices used in the characterisation of the winter barley grain mycobiome.

Index	Formula
Relative frequency	RF [%] = (n_i_/N_i_) × 100%
Dominance	Y = (n_i_/N_i_)fi
Species richness	S = no. of species in each variant
Margalef index	D′ = (S − 1)/lnNt
Shannon–Wiener index	$H^{\prime }=-\sum_{i=1}^{s}P_{i}lnP_{i},\;$ $P_{i}=N_{i}/N_{t}$
Dominance index	$\lambda =\sum_{i}^{s}p_{i}^{2}$

Nt stands for the number of all isolated cultures; n_i_/N_i_ for the number of isolates belonging to the i-th species/genus; fi for the frequency of occurrence of the genus; P_i_ = N_i_/N_t_.

## Data Availability

The original contributions presented in this study are included in the article. Further inquiries can be directed to the corresponding authors.

## Supplementary Materials

The following supporting information can be downloaded at: https://www.mdpi.com/article/10.3390/toxins18040171/s1, Table S1: Weather conditions during the growing season 2018/2019; Table S2: Weather conditions during the growing season 2019/2020; Table S3: Fertilisation and plant protection treatments in winter barley cultivation during the 2018/2019 season; Table S4: Fertilisation and plant protection treatments in winter barley cultivation during the 2019/2020 season.
