# Supplementary material for: Pre-Harvest Agronomic Reduction in Fusarium Mycotoxins in Winter Barley: Effects of Agrotechnical Intensity on Grain Mycobiome, DON/ZEN and Feed-Quality Traits
Source: Toxins (Basel). 2026 Apr 2;18(4):171. doi: 10.3390/toxins18040171 (PMC13120009; doi:10.3390/toxins18040171)
Supplement: Supplementary file 1 [file toxins-18-00171-s001.zip › toxins-4174676-supplementary.pdf]

Table S1. Weather conditions during the growing season 2018/2019.

Growing season	2018/2019										
Month / Days	Sep	Oct	Nov	Dec	Jan	Feb	Mar	Apr	May	Jun	Jul
**Air temperature (°C)**											
1 – 10	18.2	10.7	11.1	3.54	0.75	1.83	6.65	10.5	9.54	20.9	17.7
11 – 20	17.6	12.6	4.3	-0.04	0.99	4.07	6.06	8.4	11.6	23.0	17.6
21 – until the end of the month	11.6	8.2	0.97	4.1	-2.65	3.79	7.24	13.5	14.8	22.3	22.4
Monthly average	15.8	10.4	5.4	2.6	-0.91	3.19	6.7	10.8	12.1	22.1	19.3
Average of years 1986-2015	13.7	9.1	4.3	0.6	-0.4	0.6	3.8	8.9	14.4	17.3	19.6
**Precipitation (mm)**											
1 – 10	10.2	1.2	9.6	11.0	26.0	16.6	10.3	0.0	9.4	1.3	3.2
11 – 20	11.5	2.8	3.4	8.5	26.3	2.7	8.4	5.2	20.6	4.2	1.5
21 – until the end of the month	16.7	41.3	1.7	18.5	3.9	8.4	3.8	19.0	46.8	21.5	39.8
Monthly total	38.4	45.3	14.7	38.0	56.2	27.7	22.5	24.2	76.8	27.0	44.5
Total over the years 1986-2015	44.9	33.7	36.6	36.3	34.9	27.9	38.2	33.6	54.1	67.4	78.9

Table S2. Weather conditions during the growing season 2019/2020.

Growing season	2019/2020										
Days \ Month	Sep	Oct	Nov	Dec	Jan	Feb	Mar	Apr	May	Jun	Jul
**Air temperature (°C)**											
1 – 10	16.3	10.1	7.8	2.4	2.3	4.8	5.5	8.1	11.3	15.6	20.0
11 – 20	13.0	13.9	7.2	3.6	2.5	5.9	7.4	8.7	11.2	20.0	18.3
21 – until the end of the month	13.9	9.9	6.2	3.6	1.3	4.9	2.9	11.8	12.3	19.9	20.1
Monthly average	14.4	11.3	7.1	3.0	2.0	5.2	5.3	9.5	11.6	18.5	19.5
Average of years 1986-2015	13.7	9.1	4.3	0.6	-0.4	0.6	3.8	8.9	14.4	17.3	19.6
**Precipitation (mm)**											
1 – 10	34.4	7.6	4.7	3.1	6.2	26.3	7.1	0.0	20.6	29.3	20.0
11 – 20	3.9	3.2	28.2	1.6	0.0	5.3	7.9	1.5	34.1	56.6	29.1
21 – until the end of the month	3.7	7.3	1.6	10.4	4.9	31.3	4.0	4.9	22.5	8.6	4.1
Monthly total	42.0	18.1	34.5	15.1	11.1	62.9	19.0	6.4	77.2	94.5	53.2
Total over the years 1986-2015	44.9	33.7	36.6	36.3	34.9	27.9	38.2	33.6	54.1	67.4	78.9

Table S3. Fertilisation and plant protection treatments in winter barley cultivation during the 2018/2019 season.

Date	Grownig phase	Fertiliser or active substance	Dose per 1 ha
1 October 2018	Pre-sowing	Triple superphopsphate **A1+A2**	50 kg
1 October 2018	Pre-sowing	Potassium salt **A1+A2**	80 kg
1 October 2018	Pre-sowing	Seed dressing **A1+A2** tebuconazole	150g/100 kg of grain
22 October 2018	3rd leaf / beginning of tillering	Herbicide **A1+A2** chlorotoluron	2 l
4 March 2019	Beginning of stem elongation	Ammonium nitrate **A1**	50 kg
11 April 2019	1st elbow	Ammonium nitrate **A1**	10 kg
4 March 2019	Beginning of stem elongation	Ammonium nitrate **A2**	60 kg
11 April 2019	1st elbow	Ammonium nitrate **A2**	40 kg
18 April 2019	1st elbow	Growth regulator **A2** trinexapac ethyl	0.8 l
28 April 2019	3rd elbow	Mutli-micronutrient foliar fertiliser **A2**	2 l
30 April 2019	3rd elbow	Fungicide **A2** chlorothalonil	1.0 l
7 May 2019	Ear emergence	Fungicides **A2** azoxystrobin + tebuconazole	0.6 l + 0.75 l

A1 – basic agrotechnical level; A2 – intensive agrotechnical level.

Table S4. Fertilisation and plant protection treatments in winter barley cultivation during the 2019/2020 season.

Date	Grownig phase	Fertiliser or active substance	Dose per 1 ha
26 September 2019	Pre-sowing	Triple superphopsphate **A1+A2**	50 kg
26 September 2019	Pre-sowing	Potassium salt **A1+A2**	80 kg
27 September 2019	Pre-sowing	Seed dressing **A1+A2** tebuconazole	150 g/100 kg of grain
25 October 2019	3rd leaf / beginning of tillering	Herbicide **A1+A2** (chlorotoluron + diflufenican)	2 l
5 March 2020	Beginning of stem elongation	Ammonium nitrate **A1**	50 kg
27 March 2020	1st elbow	Ammonium nitrate **A1**	10 kg
5 March 2020	Beginning of stem elongation	Ammonium nitrate **A2**	60 kg
27 March 2020	1st elbow	Ammonium nitrate **A2**	40 kg
9 April 2020	1st elbow	Fungicides **A2** prochloraz + tebuconazole	1.0 l + 1.0 l
27 April 2020	3rd elbow	Growth regulators **A2** trinexapac ethyl + chlormequat chloride	0.5 l + 0.8 l
4 May 2020	3rd elbow	Mutli-micronutrient foliar fertiliser **A2**	2 l
18 May 2020	Ear emergence	Fungicides **A2** azoxystrobin + (propiconazole + cyproconazole)	0.6 l + 0.4 l

A1 – basic agrotechnical level; A2 – intensive agrotechnical level; parentheses () indicate two active substances contained within a single formulation.
